# Short message service phone reminder as an important tool to reduce absenteeism for atendance at healthcare appointments

**DOI:** 10.1186/1758-5996-7-S1-A258

**Published:** 2015-11-11

**Authors:** Mirian Farias, Daniel Klug, Balduino Tschiedel, Matilde Gerchman, Marcia Puñales

**Affiliations:** 1 Hospital da Criança Conceiçao e Instituto da Criança Com Diabetes-GHC, Porto Alegre, Brazil

## Background

Absenteeism, or missing scheduled appointments is a serious problem to the healthcare system, affecting healthcare institutions that cannot maximize their staff appointments, thus increasing their costs, and also to patients, who face difficulties in accessing the system.

## Objective

The aim of this study was to assess whether sending scheduled appointment date reminders through Short Message Service (SMS) for attendance at a healthcare unit would reduce the absenteeism in our institution.

## Materials and methods

The study enrolled patients who scheduled appointments with an interdisciplinary team (up to 5 scheduled appointments per shift), from January-December 2012, before the SMS reminders, and compared to January-December of 2013 and 2014. Absenteeism was analyzed by the total number of patients scheduled appointments (TSA), compared to the absentees at the appointed date and also the number of health care providers scheduled appointments (NSA) compared to the team's idle time during the study period. The SMS was sent a week before the appointment date.

## Results

In 2012, before the SMS intervention, TSA absenteeism was 20.0±3.3% (7736 scheduled patients/1524 no-show patients), in 2013 was 15.9±2.1% (7509/1199) and in 2014 was 15.4±1.8% (7285/1118). A TSA absenteeism reduction of 20.5% (p=0.0024) was observed in the first year of the intervention and of 23.0% (0.0004) in the second year. The NSA absenteeism in 2012 was 14.9±1.9% (17196 scheduled appointments/2510 idle time), in 2013 was 11.9±1.9% (16674/1988) and in 2014 was 11.5±1.7% (17867 /2024). During the study period, there was an idleness reduction of TSA (2012: 127±14.7, 2013: 99.9±19.0 and 2014: 93.2±17.9, p=0.0003) and NSA (2012: 209.2±26.6, 2013: 165.7±41.9 and 2014: 168.7±31.4, p=0.0052).

## Conclusion

The use of phone reminders with scheduled appointment dates through SMS was effective in reducing healthcare absenteeism, being an important tool to optimize the services offered by the institution.

